# A dataset of ITS-G5 and cellular vehicular connectivity in urban environment

**DOI:** 10.1016/j.dib.2023.109846

**Published:** 2023-11-26

**Authors:** Duarte Dias, Rodrigo Rosmaninho, Andreia Figueiredo, Pedro Almeida, Miguel Luís, Pedro Rito, Duarte Raposo, Susana Sargento

**Affiliations:** a Instituto de Telecomunicações, 3810-193 Aveiro, Portugal; b Departamento de Eletrónica, Telecomunicações e Informática, Universidade de Aveiro, 3810-193 Aveiro, Portugal; c Instituto Superior Técnico, Universidade de Lisboa, Av. Rovisco Pais 1, 1049-001 Lisboa, Portugal

**Keywords:** Vehicular networks, Vehicle-to-infrastructure, ETSI ITS-G5, LTE/5G

## Abstract

Connecting vehicles to the Internet is an emerging challenge of wireless networks. There are two competing methods for achieving this. First, the wireless local area network (WLAN) approach is based on the IEEE 802.11p standard (in its European version called ETSI ITS-G5) created for Cooperative-Intelligent Transportation System applications. Second, the cellular network approach is based on LTE/5G technologies which have been exploited in recent years to support vehicular applications. Advantages such as high bandwidth, high coverage and high reliability make cellular networks a great option for the vehicular environment.

This article describes two datasets that support the analysis of WLAN (ETSI ITS-G5) and Cellular (LTE/5G) technologies in a real vehicular and road environment. The two datasets summarize the results obtained in a collection of network performance tests performed in the city of Aveiro, Portugal. In these tests, a set of vehicles (8 On-Board Units) moved randomly around the city, passing near a group of stationary nodes (11 Road-Side Units) uploading data to a server. In the WLAN dataset, data was sent using the ETSI ITS-G5 technology, whereas, in the Cellular dataset, data was sent using LTE/5G technologies. While testing, location, signal quality, and network performance data (achieved throughput, jitter, etc.) were collected.

This dataset can support a realistic analysis of WLAN and Cellular performance in an environment that is not only vehicular but also urban, with obstacles and interference.

Specifications Table

**Table:** 

Subject	Computer Networks and Communications
Specific subject area	Vehicular wireless networks
Data format	Filtered
Type of data	Table
How the data were acquired	Pseudorandom data was sent from multiple mobile nodes (clients) to a server using the iPerf3 tool. Tests were performed with both ETSI ITS-G5 and cellular technologies. Mobile nodes were responsible for collecting GPS and signal quality data for each technology. Regarding the server, it was responsible for obtaining network performance metrics. Data from both sides were merged using the timestamp. Hardware used: (i) PC Engines APU2 platforms [1] (used as both stationary and mobile nodes), running the Debian 11 Operating System; (ii) Mobile Mark MAG6-5900/1575 antenna [2], installed on both mobile and stationary nodes.
Data collection	Multiple mobile nodes (clients), called On-Board Units (OBUs), move around the city, sending data to a server located in Instituto de Telecomunicações de Aveiro. Regarding WLAN, the data is sent via multiple stationary city nodes. The nodes are part of the Aveiro Tech City Living Lab platform [3], which includes a vast set of nodes, called Road-Side Units (RSUs), strategically spread across the city. Regarding Cellular, the data is sent via a cellular base station. The mobile nodes made multiple random paths around the Road-Side Units. Mobile client speeds varied between 0 and 50 Km/h.
Data source location	City/Town/Region: Aveiro Country: Portugal
Data accessibility	Repository name: Zenodo DOI: 10.5281/zenodo.10083808 Direct URL to data: https://doi.org/10.5281/zenodo.10083808

## 1 Value of the Data

• **Importance:** The data provided shows the behaviour of WLAN and Cellular in a vehicular context. These are datasets of some importance since they express the behaviour of the two technologies in a real city environment. This type of environment, due to the requirement of a deployed infrastructure in the case of WLAN (ETSI ITS-G5), makes this data difficult to obtain.
• **Target audience:** The presented datasets, specifically the WLAN, provide utility to researchers in the vehicular wireless communications area. It is especially relevant for those who lack the resources/infrastructure to conduct their vehicular experiments or to compare their results with those of others.
• **Future use:** The datasets presented here were collected to support a comparative analysis of the solutions in a vehicular and road environment. A possible applicability of this data could be in the creation of prediction models based on machine learning algorithms.

## 2 Data Description

The first dataset contains data related to the WLAN (ETSI ITS-G5) technology. It consists of data obtained between September 2022 and March 2023. It was collected by multiple vehicles in multiple collection periods, not necessarily all simultaneously. The routes taken by the vehicles were random and restricted to the city of Aveiro. Every entry in the dataset corresponds to a 100-millisecond iPerf3 measurement.

**Fig. 1:**
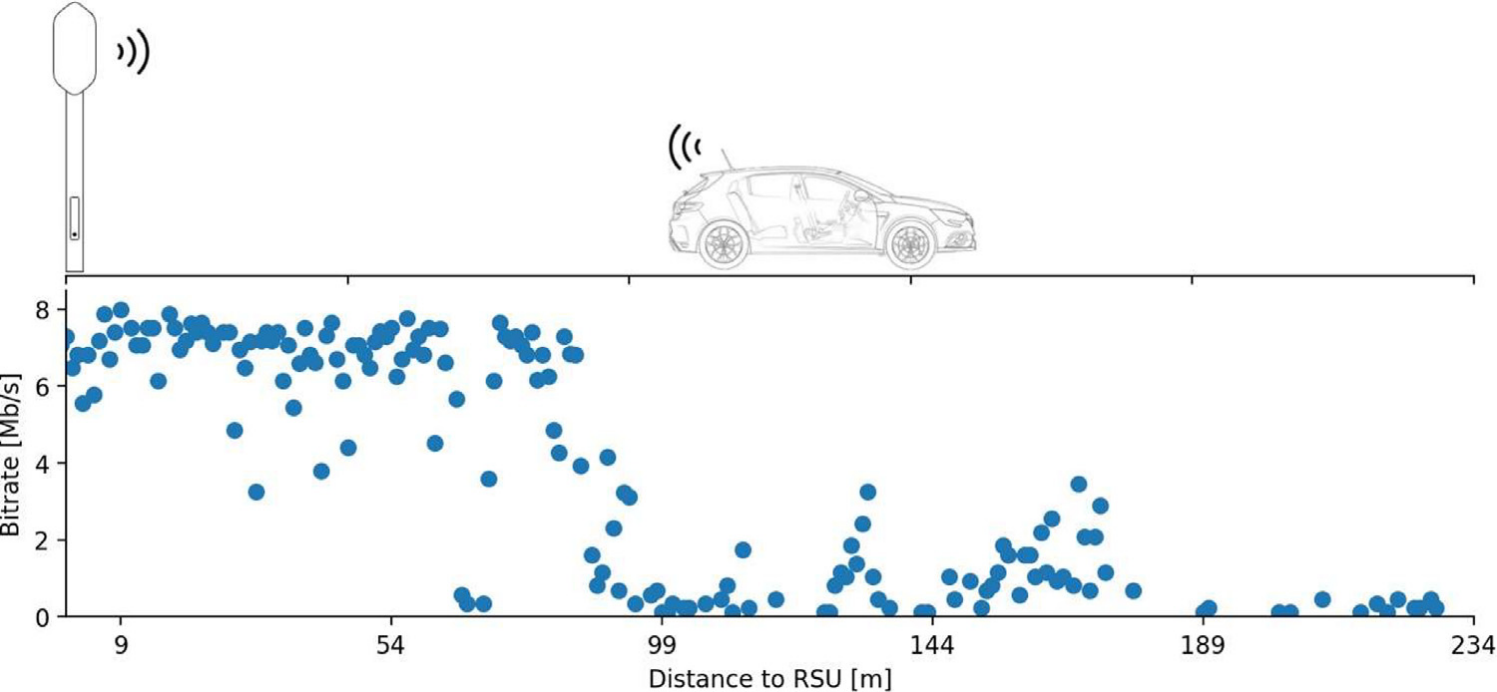
Small segment of the collected data.

This dataset contains only one file that includes both location and network performance data:

• **“data_wlan.csv”**: The file contains network performance metrics associated with location and signal quality data. Information regarding the vehicle and the RSUs used are also present. Table 1 shows all file attributes with their description and respective value if missing. In some tests, certain metrics were left unmeasured. To address this, any absent values were replaced with the corresponding value found in the "Value if missing'' section. Fig. 1 depicts a small segment of the collected data in the form of a graph/schematic, showcasing the data's behaviour.

**Table 1:** ITS-G5 attributes description.

Attributes	Description	Value if missing
obu_id	ID of the OBU that obtained the data.	–
rsu_id	ID of the RSU used to obtain the data.	–
lat	Latitude geographical coordinate of the OBU.	–
long	Longitude geographical coordinate of the OBU.	–
head	Heading indicates the direction of the OBU (in degrees).	–
dist_rsu	Distance between OBU and RSU (in meters).	–
rssi_rsu	Received Signal Strength Indicator perceived by the RSU (in dBm).	−32,768.0
rssi_obu	Received Signal Strength Indicator perceived by the OBU (in dBm).	−32,768.0
bitrate	Bitrate (in Mb/s).	–
jitter	Jitter (in ms).	–
loss	Packet loss (in%).	–
rtt	Round-Trip Time (in ms).	−1

The second dataset contains data related to the cellular technologies. It consists of data obtained between March and April 2023. It was collected by one vehicle in multiple collection periods. The routes taken by the vehicle were random and restricted to the district of Aveiro. Every entry in the dataset corresponds to a 100-millisecond iPerf3 measurement.

This dataset contains only one file that includes both location and network performance data:

• “**data_cellular.csv”:** The file contains network performance metrics associated with location and signal quality data. Information regarding the vehicle is also present. Table 2 shows all file attributes with their description and respective value if missing. In some tests, certain metrics were left unmeasured. To address this, any absent values were replaced with the corresponding value found in the “Value if missing” section.

**Table 2:** Cellular attributes description.

Attributes	Description	Value if missing
obu_id	ID of the OBU that obtained the data.	–
lat	Latitude geographical coordinate of the OBU.	–
long	Longitude geographical coordinate of the OBU.	–
head	Heading indicates the direction of the OBU (in degrees).	–
tech	Cellular technology in use (lte or 5g).	–
cid	Cell ID.	–
rsrp_lte	Reference Signal Received Power in LTE (in dBm).	−32,768.0
rsrq_lte	Reference Signal Received Quality in LTE (in dB).	−32,768.0
snr_lte	Signal to Noise Ratio in LTE (in dB).	−32,768.0
rsrp_5g	Reference Signal Received Power in 5G (in dBm).	−32,768.0
rsrq_5g	Reference Signal Received Quality in 5G (in dB).	−32,768.0
ser_5g	Signal to Noise Ratio in 5G (in dB).	−32,768.0
bitrate	Bitrate (in Mb/s).	–
jitter	Jitter (in ms).	–
loss	Packet loss (in%).	–
rtt	Round-Trip Time (in ms).	−1

## 3 Experimental Design, Materials and Methods

The two datasets presented here were collected to show the performance of both the ETSI ITS-G5 and the Cellular technologies in a realistic mobility environment within the city. For this purpose, both the Road-Side Units infrastructure and a set of mobile nodes (8 in total) were used. While the mobile nodes were on their journeys, they transmitted data to a server situated at the Instituto de Telecomunicações in Aveiro, Portugal. The iPerf3 tool was responsible for generating the transmitted data, showcasing its capability to accurately simulate traffic at the packet level by replicating relevant stochastic processes. Following each test, the tool provides a collection of network metrics that convey the network's status.

**Fig. 2:**
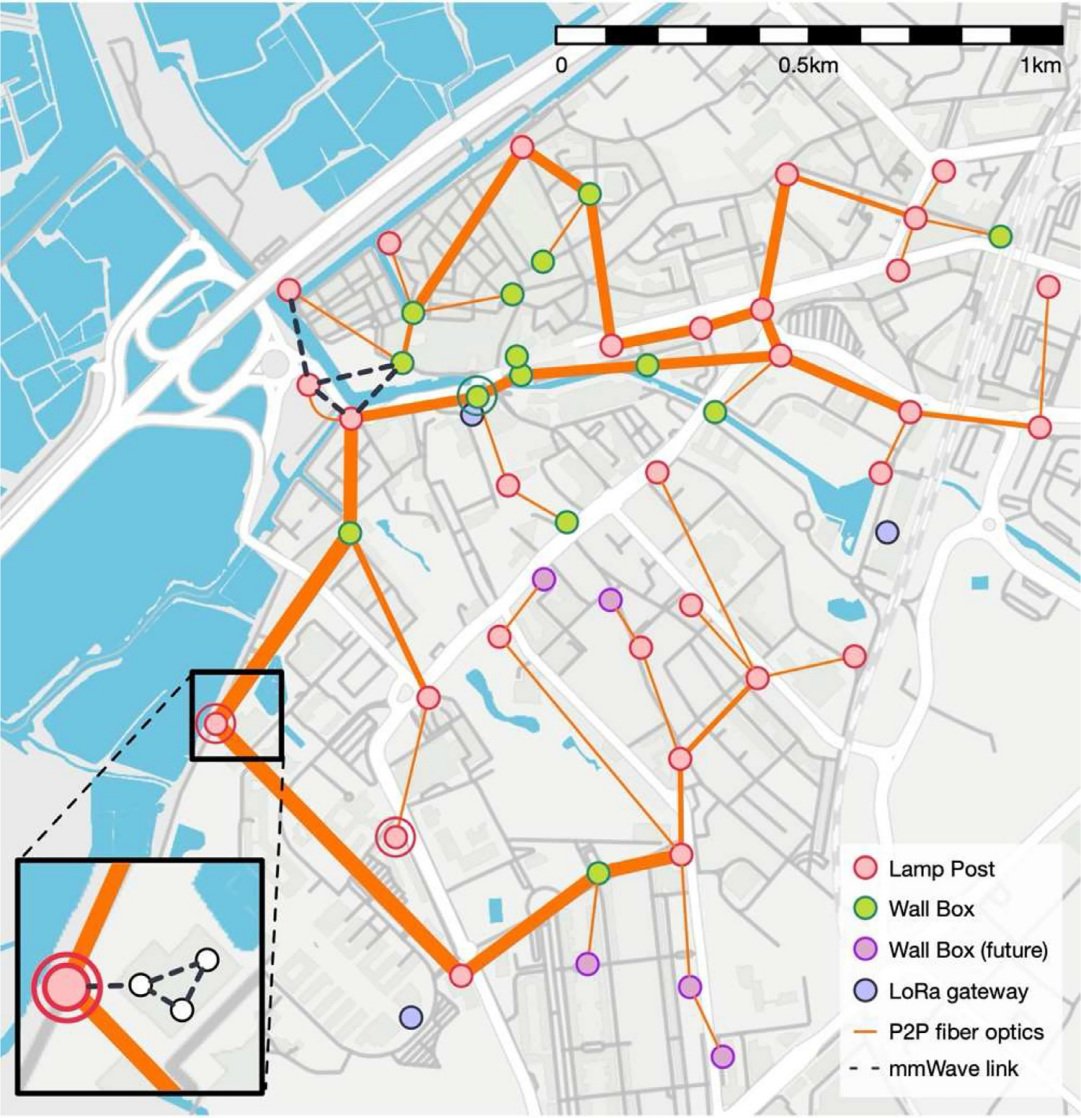
Aveiro Tech City Living Lab connectivity map [3].

The Aveiro Tech City Living Lab platform, deployed in Aveiro, Portugal, is an initiative created by the city and Instituto de Telecomunicações in Aveiro, that combines a multi-technology, advanced, large-scale communication, sensing and computation infrastructure for data management and innovative analytics. The platform comprises multiple nodes, called Road-Side Units, strategically spread across the city that are connected to a data processing centre. In Fig. 2, the platform map illustrates Road-Side Units represented by Lamp Posts and Wall Boxes. The thickness of the optical fiber links in the figure reflects the quantity of fibers traversing each segment.

These datasets were obtained based on several random paths through the city of Aveiro at different times over several months. One vehicle was assigned to gather cellular data, and the rest were responsible for obtaining WLAN (ETSI ITS-G5) data. As mobile nodes follow unpredictable paths, they may not consistently navigate near Road-Side Units. However, it allows for a typical vehicle behaviour in urban areas.

In both cases, typical road speeds were used, with a maximum of 50 km/h (the legal limit). This speed enables the collection of data in a realistic vehicular urban environment. The set of 8 vehicles circulated the city on several days for two months (early 2023) during variable periods, routes and weather conditions.

**Fig. 3:**
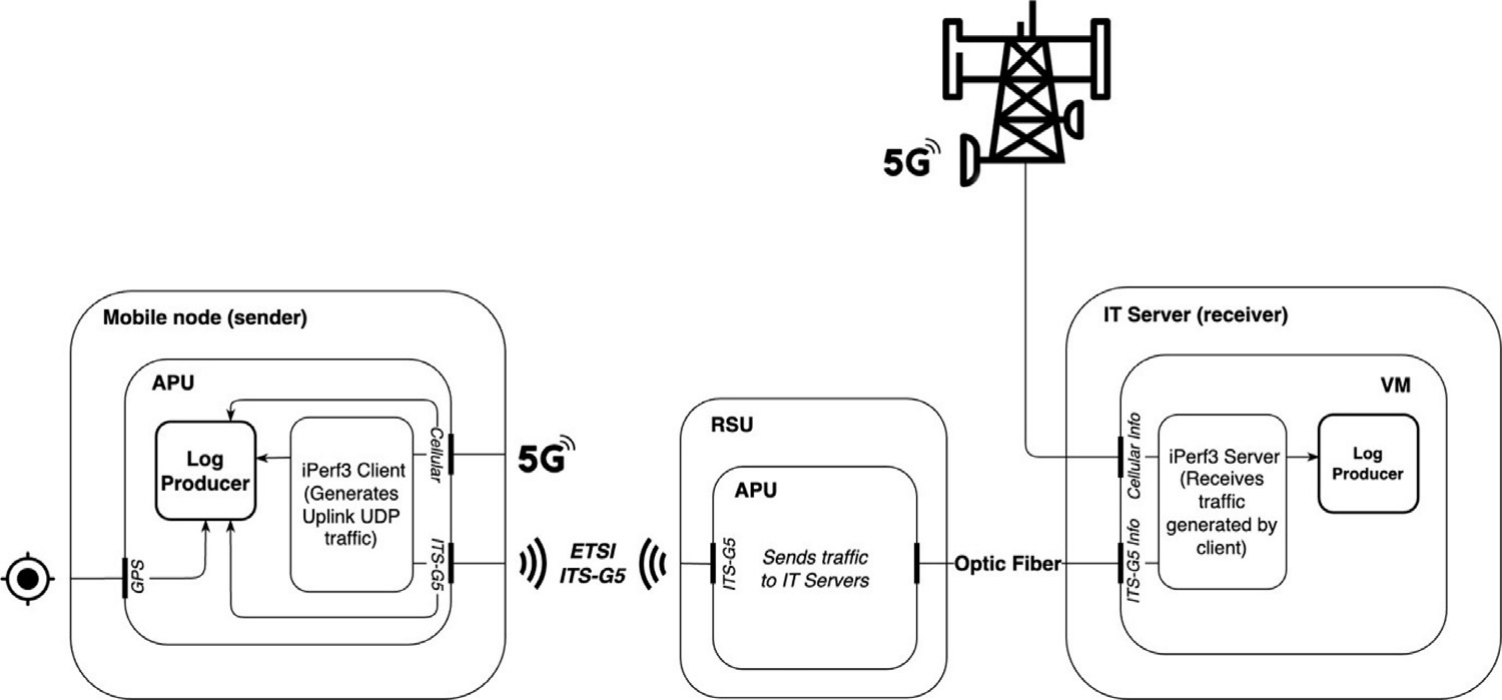
System architecture.

Regarding the software and hardware used in the experiments, these are described in Fig. 3.

The system consists of two main nodes, a sender and a receiver. There are also intermediate nodes that vary depending on the communication technology. In the context of ITS-G5, the Road-Side Units (RSUs) act as the intermediate nodes, facilitating the relay of traffic to the Virtual Machine (VM) situated at Instituto de Telecomunicações of Aveiro, functioning as a central hub. This setup connects all Road-Side Units to the central Virtual Machine where the receiver is located. Regarding cellular, the relay is carried out by the LTE/5G cellular network.

The sender consists of a PC Engines APU2 platform with ETSI ITS-G5 technology or cellular, depending on whether the vehicle is allocated to ETSI ITS-G5 or cellular tests. It is also equipped with a GPS antenna to obtain location coordinates. The Road-Side Units runs the iPerf3 tool in client mode, generating UDP traffic with a target bandwidth of 10Mb/s or 50Mb/s for the ETSI ITS-G5 and cellular tests, respectively. There is also a logging module that is responsible for aggregating the metrics acquired at 10 Hz from the iPerf3 tool. This module also saves the timestamp and GPS coordinates (this one limited to 1 Hz) so that it is possible to merge all the information later.

The use of the UDP network protocol is explained by the intermittent connection between the mobile nodes and the Road-Side Units (in the case of the ETSI ITS-G5). In most routes, the duration of connection to the Road-Side Units is reduced, which could cause problems in the control messages present in the TCP protocol. The bandwidth limitation in the case of the ETSI ITS-G5 is due to technology limitations (limited to 8–9 Mb/s in a real-world environment). For cellular, this constraint is not dictated by the technology itself but rather by the potential saturation of the cellular network in areas with limited coverage.

Regarding the receiver, instead of the modules running in an APU, these run in a dedicated Virtual Machine with the same modules as the sender. In particular, it contains a logging module and the iPerf3 tool, currently operating in server mode. Thus, this unit receives traffic generated on the sender side, both from the cellular and from the WLAN side. There is a server instance for each client (mobile node), with eight server instances in total, each with a specific port for the vehicle. The network performance metrics obtained by iPerf3 are stored with an associated timestamp by the logging module. The data is obtained with a frequency of 10 Hz and saved at this frequency.

The data obtained from both sides (sender and receiver) are then merged based on the timestamp of the measurements. In this way, it is possible to combine the information. One of the facts to consider is that the GPS information is collected at a frequency of 1 Hz, with the rest at 10 Hz. In this case, data interpolation is performed to obtain intermediate coordinate points without compromising the accuracy of the information. Another point to consider is the synchronization of the clocks of both the sender and the receiver. This synchronization is done at the beginning of each test for each vehicle and ensures that the information can be merged correctly. Thereby, it is possible to combine the information to create these two datasets.

As this is a continuous collection of data, in periods of non-connectivity (high intermittence), the values of the network metrics come with null values. Since this data is of little relevance, it was decided to remove these values from both datasets. In the case of cellular, as the connection is very stable, there are few null values.

**Fig. 4:**
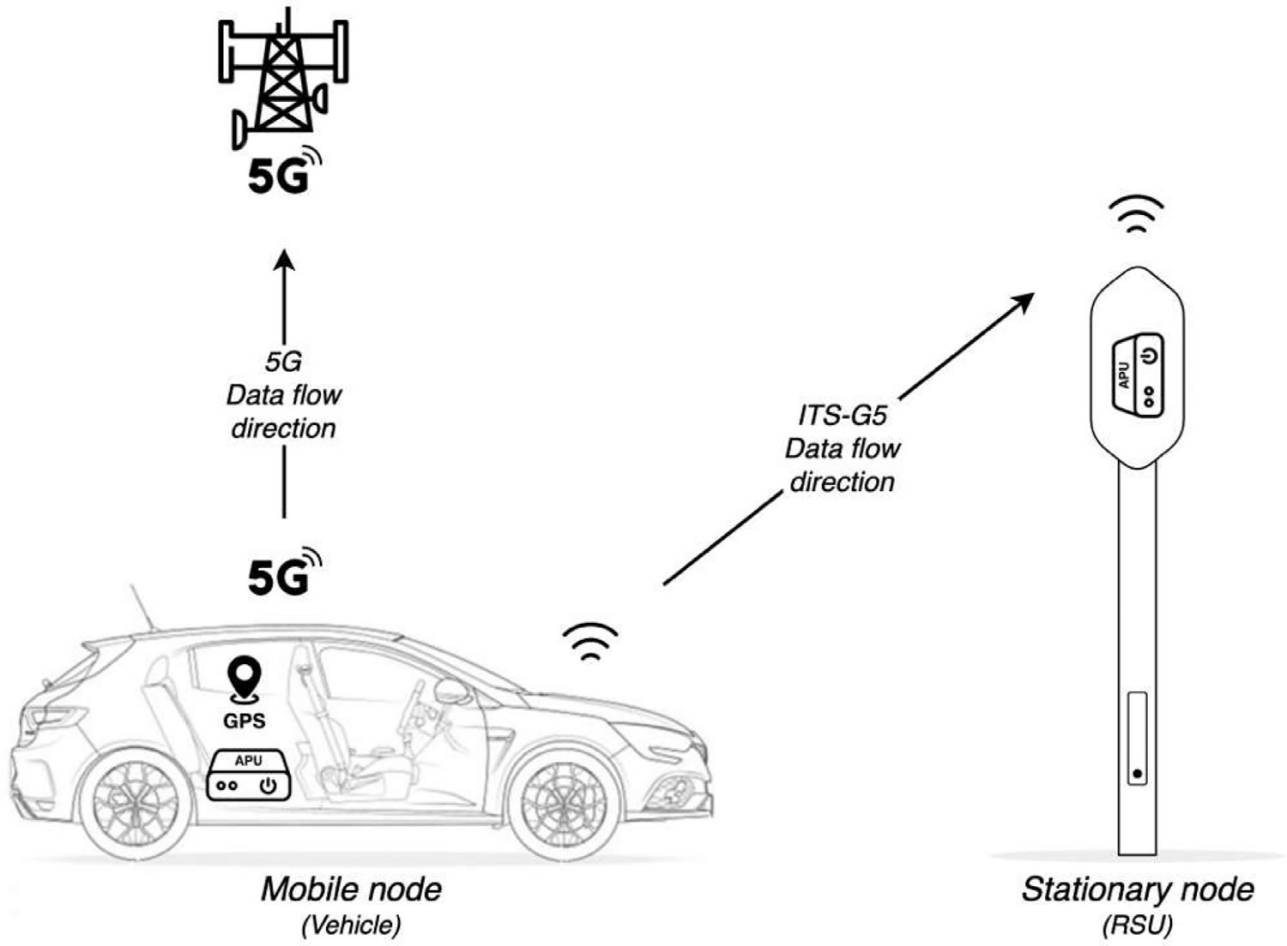
Data collection setup.

Concerning the physical setup of the described system, it is shown in Fig. 4. Regarding the mobile nodes, a fleet of vehicles was employed. As for static nodes, Road-Side Units from the Aveiro Tech City Living Lab platform were used. Regarding the communication devices used in the vehicles, these were placed on the roof to reduce obstructions as much as possible. In the case of Road-Side Units, the communication devices are inside the upper dome of the pole, with only a slight blockage of the pole's protection.

## Limitations

None.

## Ethics Statement

The work here presented did not involve human subjects, animal experiments, or any data collected from social media platforms.

## CRediT authorship contribution statement

**Duarte Dias:** Conceptualization, Methodology, Software, Validation, Resources, Data curation, Writing – original draft, Visualization. **Rodrigo Rosmaninho:** Software, Resources. **Andreia Figueiredo:** Software, Resources. **Pedro Almeida:** Software, Resources. **Miguel Luís:** Conceptualization, Methodology, Validation, Writing – review & editing, Funding acquisition. **Pedro Rito:** Conceptualization, Methodology, Validation, Writing – review & editing. **Duarte Raposo:** Conceptualization, Methodology, Validation, Writing – review & editing. **Susana Sargento:** Conceptualization, Methodology, Validation, Writing – review & editing, Funding acquisition.

## Data Availability

ATCLL Network Performance Data (Original data) (Zenodo)
